# Genetic Evaluation of Water Use Efficiency and Nutrient Use Efficiency in *Populus deltoides* Bartr. ex Marsh. Seedlings in China

**DOI:** 10.3390/plants13162228

**Published:** 2024-08-11

**Authors:** Chengcheng Gao, Chenggong Liu, Cun Chen, Ning Liu, Fenfen Liu, Xiaohua Su, Qinjun Huang

**Affiliations:** 1 State Key Laboratory of Tree Genetics and Breeding, Research Institute of Forestry, Chinese Academy of Forestry, Beijing 100091, China; gaocc@caf.ac.cn (C.G.); liucgwlq@caf.ac.cn (C.L.); ningliu666666@gmail.com (N.L.); lfflff0122@163.com (F.L.); suxh@caf.ac.cn (X.S.); 2 Key Laboratory of Tree Breeding and Cultivation, State Forestry and Grassland Administration, Beijing 100091, China; 3 School of Life Sciences, Qilu Normal University, Jinan 250013, China; chencun0610@163.com; 4 UGent-Woodlab (Laboratory of Wood Technology), Department of Environment, Ghent University, 9000 Ghent, Belgium

**Keywords:** poplar, genetic variation, δ^13^C and δ^15^N, water and nutrient use efficiency

## Abstract

*Populus deltoides* Bartr. ex Marsh. represents a valuable genetic resource for fast-growing plantations in temperate regions. It holds significant cultivation and breeding potential in northern China. To establish an efficient breeding population of poplar, we studied the genetic variation of *P. deltoides* from different provenances. Our focus was on genotypes exhibiting high growth rates and efficient water and nutrient use efficiency (WUE and NUE). We evaluated 256 one-year-old seedlings from six provenances, measuring height, ground diameter, total biomass, and leaf carbon and nitrogen isotope abundance (δ^13^C and δ^15^N). Our analytical methods included variance analysis, multiple comparisons, mixed linear models, correlation analysis, and principal component analysis. The results showed that the coefficient of variation was highest for δ^15^N and lowest for δ^13^C among all traits. Except for δ^15^N, the effects of intra- and inter-provenance were highly significant (*p* < 0.01). The rates of variation for all traits ranged from 78.36% to 99.49% for intra-provenance and from 0.51% to 21.64% for inter-provenance. The heritability of all traits in AQ provenance was over 0.65, and all exhibited the highest level except for seedling height. All traits were significantly positively correlated with each other (*p* < 0.05), while ground diameter, total biomass, and WUE were highly significantly negatively correlated with latitude (*p* < 0.01). After a comprehensive evaluation, two provenances and eight genotypes were selected. The genetic gains for seedling height, ground diameter, total biomass, WUE, and NUE were 27.46 cm (178-2-106), 3.85 mm (178-2-141), 16.40 g (178-2-141), 0.852‰ (LA05-N15), and 3.145‰ (174-1-2), respectively. Overall, we revealed that the abundant genetic variation in *P. deltoides* populations mainly comes from intra-provenance differences and evaluated provenances and genotypes. The results of this study will contribute to optimizing and enhancing the breeding process of Chinese poplar and improving the productivity of fast-growing plantations.

## 1. Introduction

Poplar is one of the fastest-growing tree species in temperate regions, playing a significant role in the production of wood raw materials, the supply of bioenergy, and ecological protection [1,2], and is one of the major species cultivated in plantations around the world [3]. China boasts the largest area of poplar plantation forests globally, covering 8.5 million hectares, which accounts for about one-third of the world’s poplar plantations [4]. It plays an irreplaceable and important role in the construction of China’s national reserve forests, the creation of farmland protection forests, and the maintenance of domestic timber production. *Populus deltoides* Bartr. ex Marsh., as one of the most suitable tree species for the intensive management of industrial timber with short rotation periods in the mid-latitude regions of the world, also has a dominant position in the poplar plantations in China [5]. However, *P. deltoides* is highly sensitive to drought and nutrient stress, which significantly limits its effectiveness in the cultivation of poplars in the arid, infertile, saline, and sandy regions of North China. In addition, as a common parent for hybrid selection, the existing resources of *P. deltoides* in China are few, and all of them are imported from abroad [6]. Therefore, evaluating the physiological characteristics of the existing *P. deltoides*, such as drought resistance and tolerance to nutrient deficiency, as well as selecting germplasm resources with superior traits, has become crucial for the breeding of this poplar species at the present stage.

Throughout the life cycle of plants, environmental stresses can significantly impact their growth and development [7]. In particular, the participation of water and nitrogen is indispensable for various metabolic activities and physiological reactions [8,9]. Optimal levels of water and nitrogen are crucial for enhancing plant growth and photosynthetic capacity [10], and the effective supply of water and nitrogen is the core element for improving plant primary productivity [9,11]. With the effects of global warming and human activities in recent years, the global landmass is generally heading towards increased aridity [12,13,14], which inevitably leads to greater evaporative loss of soil–water from otherwise arid regions, and also exacerbates the degree of mineralization of soils, which in turn restricts plant survival and growth [15]. Providing adequate water and applying nitrogen fertilizer can significantly enhance the growth and yield of crops such as maize (*Zea mays* L.) [16], wheat (*Triticum aestivum* L.) [17], cotton (*Gossypium hirsutum* L.) [18], and others [19,20]. However, for fast-growing, short-rotation tree species, which tend to have higher water and nutrient requirements, frequent harvesting can limit nutrient cycling and reduce soil fertility [21,22,23]. Additionally, the excessive use of nitrogen fertilizers can in turn lead to reduced yield, quality, and nutrient effectiveness, as well as water, air, and soil pollution [20,24,25]. Therefore, in a resource-limited environment, the selection and breeding of plant material that uses resources efficiently is an important method of attaining sustainable forest development.

The pattern of plant uptake and utilization of water and nutrients determines, to a certain extent, the outcome of plant responses to changes in environmental water status [26]. Water use efficiency (WUE) and nitrogen use efficiency (NUE) are widely used to assess the water and nutrient utilization status of plants and are key physiological parameters reflecting the relationship between plant productivity and water and nutrient utilization [27,28]. However, traditional research methods for evaluating water and nutrient utilization often face limitations due to their destructive nature or restrictive conditions [29]. With the development of testing methods such as mass spectrometry, the isotope natural abundance method has become a new technical tool to quantitatively study plant WUE and NUE due to its advantages of being unlimited in time and space, non-invasive, and easy to measure [30,31,32,33]. In C_3_ plants, the abundance of carbon stable isotope (δ^13^C), which is primarily associated with the ratio of leaf internal to atmospheric CO_2_ concentration (C_i_/C_a_), serves as a reliable indicator of long-term internal WUE [34]. Nitrogen stable isotope abundance (δ^15^N) can detect and quantify plant N inputs and losses and is used to characterize plant NUE [35,36]. Currently, δ^13^C and δ^15^N are widely used in crop and forest breeding studies [37,38,39,40,41]. However, studies on forest trees have primarily concentrated on investigating the effects of artificially controlled environmental factors on WUE or NUE, including drought stress [42], salinity stress [43], and nutrient addition [44]. Chinese researchers have studied the WUE and NUE physiological mechanisms of *P. tomentosa* Carrière [45], *P.* × *canadensis* Moench [46], and *P. alba* L. × *P. glandulosa*) [47]. Nevertheless, current poplar research in China has not adequately addressed the genetic variation characteristics of provenances, which limits the selection of breeding parents with high WUE and NUE, thereby hindering the extension of poplar cultivation to more arid and barren regions.

*P. deltoides* originates from the lower Mississippi River in North America and has a natural distribution from southern Canada to the southeastern United States [5,48], and was introduced to China for the first time in the 1950s [49]. Currently, Chinese researchers are selecting and breeding several genotypes of *P. deltoides* with improved traits, successfully propagating them in China, but the number of bred cultivars is still insufficient, and most of the cultivars are mainly cultivated in the southern region, where precipitation is plentiful and soil fertility is relatively high [50]. In regions characterized by short growing seasons, low precipitation, and low soil fertility, there are limited suitable cultivars of *P. deltoides*, resulting in generally low-yielding stands and a low application rate of cultivars [51]. This situation constrains the yield of Chinese poplar wood, making efficient poplar breeding an urgent and significant task in contemporary research. Building on the core germplasm construction previously established by our research team [52], this study aims to explore the genetic variation patterns of WUE and NUE among provenances of *P. deltoides*. We hope to obtain breeding materials of *P. deltoides* for arid and semi-arid regions in northern China. The results will also address the following key questions: Is there significant genetic variation in growth, WUE, and NUE among provenances? Which provenance exhibits higher heritability? Is there consistency in growth, WUE, and NUE? What are the best provenances and genotypes? How much genetic gain can we achieve?

## 2. Results

### 2.1. Genetic Variation in Growth and Biomass

Table 1 shows that at inter-provenance level, seedling height, ground diameter and total biomass ranged from 61.56 cm to 77.67 cm (mean: 68.12 cm), 7.02 mm to 8.20 mm (mean: 7.48 mm), and 19.10 g to 23.28 g (mean: 20.65 g), respectively. Seedling height was maximum in AW, ground diameter, and total biomass in AL. The mean CV for seedling height, ground diameter, and total biomass was 22.48%, 13.57%, and 27.11%, respectively. The CVs across different provenance sources ranged from 17.23 % to 29.91%, 9.54% to 17.89%, and 18.91% to 31.92%, respectively. There were large variations in seedling height, ground diameter, and total biomass at the provenance level, with total biomass > seedling height > ground diameter in the order of variability.

Table 2 shows that there were highly significant differences (*p* < 0.01) in plant height, ground diameter, and total biomass in intra- and inter-provenance, with intra-provenance variation accounting for 90.53%, 83.48%, and 93.75%, respectively, which were higher than that of inter-provenance variation. Furthermore, seedlings from AW exhibited greater height, while AL showed significantly a larger ground diameter and total biomass compared to other provenances.

### 2.2. Genetic Variation in Leaf δ^13^C and δ^15^N

The results show (Table 3) that at the inter-provenance level, the variability of δ^13^C ranged from −30.555‰ to −29.804‰ (average: −30.164‰) and δ^15^N ranged from −0.967‰ to −0.656‰ (average: −0.831‰), with δ^13^C and δ^15^N being the largest in AI. The average CVs of δ^13^C and δ^15^N were 2.11% and 94.9%, respectively, with ranges of 1.81% to 2.48% and 85.54% to 114.89%.

Table 4 shows that leaf δ^13^C was significantly different at intra- and inter-provenance levels (*p* < 0.01), while leaf δ^15^N was significantly different at intra-provenance level (*p* < 0.01), but not at inter-provenance level (*p* < 0.05). The percentage of variation in δ^13^C and δ^15^N at intra-provenance level was 78.36% and 99.49%, respectively, which were all higher than inter-provenance level. Additionally, the leaf δ^13^C of AI, AL, and AT provenances were significantly higher than those of the provenances, as shown in Table 3.

### 2.3. Heritability of Traits

Table 5 shows that the heritability for all traits in AQ was above 0.65 and was at the highest level for all traits in this provenance except seedling height. WUE was almost the highest heritability trait for all the provenances, except for AT, where the heritability of WUE was second only to the heritability of seedling height. The heritability for WUE was the most constant among all the traits. The lowest heritability for seedling height (0.36) was recorded for AM, and the lowest heritability for NUE was recorded for AI, AW, and AT with 0.42, 0.39, and 0.32, respectively. The lowest heritability for total biomass was recorded for AQ and AL, with 0.66 and 0.48, respectively.

### 2.4. Correlation Analysis of Parameters

**Figure 1:**
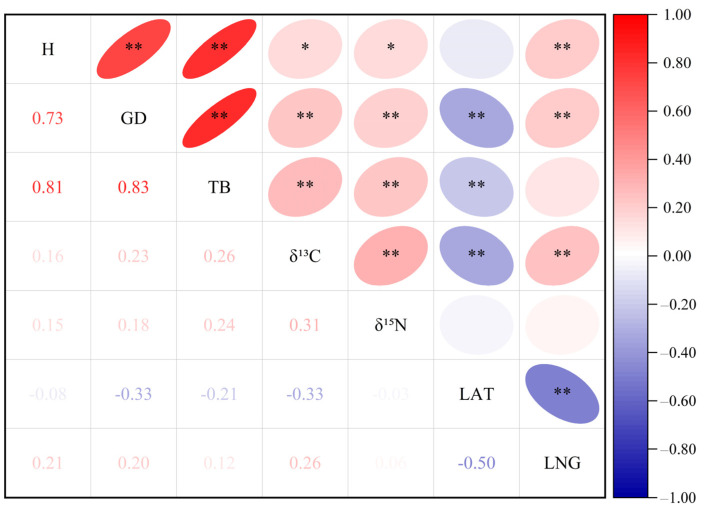
Correlation coefficient among plant traits, longitude, and latitude. The lower left corner is the correlation coefficient. The red font indicates a positive correlation and the blue font indicates a negative correlation. The color of the font indicates the strength of the correlation: the deeper the color, the stronger the correlation. The upper right corner is a highly significant level. The red indicates a positive correlation and blue indicates a negative correlation. The elliptical eccentricity size and the color depth indicate the correlation strength: the greater the elliptical eccentricity, the deeper the color, the stronger the correlation. * indicates *p* less than 0.05 and ** means *p* less than 0.01. The right color column represents the correlation coefficient. Presented here are the latitude, the longitude (LNG), the height (H), the ground diameter (GD), the total biomass (TB), the carbon isotope composition (δ^13^C), and the nitrogen isotope composition (δ^15^N).

Figure 1 shows that seedling height, ground diameter, total biomass, leaf δ^13^C, and leaf δ^15^N were significantly (*p* < 0.05) or extremely significantly (*p* < 0.01) positively correlated with one another. All traits, with the exception of seedling height and leaf δ^15^N, exhibited a highly significant (*p* < 0.01) negative correlation with LAT. With the exception of total biomass and leaf δ^15^N, the rest of the traits were extremely significantly (*p* < 0.01) positively correlated with LNG.

### 2.5. Comprehensive Evaluation

**Figure 2:**
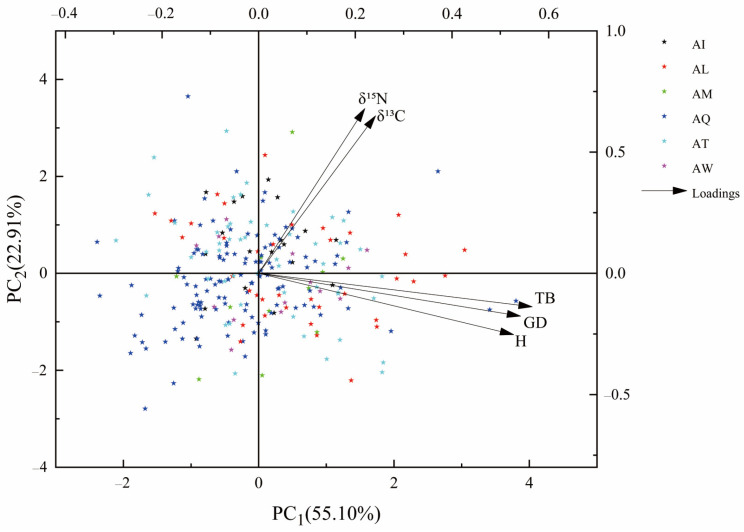
PCA analysis of 256 genotypes in 6 provenances. The x-axis and y-axis represent principal component 1 (PC_1_) and principal component 2 (PC_1_) with the proportions. Presented here are the following: height (H), ground diameter (GD), total biomass (TB), carbon isotope composition (δ^13^C), nitrogen isotope composition (δ^15^N), Iowa America (AI), Louisiana America (AL), Missouri America (AM), Tennessee America (AT), Quebec Canada (AQ), and Washington America (AW).

A total of two factors were extracted from the principal component analysis with a cumulative contribution of 78.01% (Table 6). The first principal component included the total biomass, ground diameter, and seedling height with a contribution of 55.10%, characterizing growth and total biomass. The second principal component included δ^13^C and δ^15^N with a contribution of 22.91%, characterizing WUE and NUE (Figure 2).

Combined with the results of the principal component analysis, a comprehensive evaluation model was established using the fuzzy mathematical affiliation function method. Subsequently, the genotypes were screened according to the comprehensive score, and as shown in Table 7, there were eight genotypes with superior WUE and NUE, of which five were from the AL provenance and three from the AQ provenance. Except for genotype LA05-N27, there were two or more single traits with excellent performance (Table S2). Moreover, the genetic gain of traits was calculated for each genotype and all the traits had positive genetic gain. The highest genetic gain for seedling height, ground diameter, total biomass, WUE, and NUE were 27.46 cm, 3.85 mm, 16.40 g, 0.852‰, and 3.145‰, respectively, corresponding to genotypes 178-2-106, 178-2-141, 178-2-141, LA05-N15, and 174-1-2, in that order.

## 3. Discussion

### 3.1. Genetic Variation of P. deltoides

Inheritance and variation are the basis of tree breeding and its genetic improvement, where the coefficient of variation provides an assessment of the genetic variability of a trait or population [53,54]. Generally, the level of genetic variation is categorized into three classes, namely, low (<10%), medium (10% to 20%), and high (>20%) [55]. A higher classification indicates that an individual or population possesses a greater level of genetic diversity and enhanced capacity for environmental adaptation [56,57]. In this study, the coefficients of the variation in phenotypic traits (seedling height, ground diameter, and total biomass) of 256 *P. deltoides* genotypes were all at a medium-high level, suggesting that these individuals possess diversified genetic information and a large selection potential. This result has similarity with previous findings in *Widdringtonia whteir* (Rendle) Silba [58], *Larix olgensis* A. Henry [59] and *Pinus sibirica* (Ledeb.) Turcz. [60]. In addition, the coefficient of variation for intra-provenance NUE (δ^15^N) was as high as 94.90%, which provided a material basis for selecting genotypes that are suitable for efficient nutrient utilization in soil-poor areas of northern China, thereby supporting the hypothesis posited by Villani et al. [61] that the wide distribution of a species is associated with large genetic variation among populations. However, some traits in plants are genetically conserved during evolution or are influenced by the consistency of environmental conditions and exhibit low levels of genetic variation [62]. In this study, poplars had a low level of variation in WUE (δ^13^C) (2.11%), which may be due to the fact that carbon is a basic structural substance that constitutes the plant skeleton [63] and is in high abundance as an energy source for physiological activities, such as metabolism, growth, development, and reproduction. Consequently, carbon is highly abundant in plants and exhibits low variability [64], which is consistent with the findings of Müller et al. [65].

Analysis of variance (ANOVA) is also an important method for assessing the magnitude of variation in tree breeding studies [66]. In this study, the differences in the phenotypic traits, WUE, and NUE of seedlings reached extremely significant levels across inter-provenance; except for NUE, the differences in each trait in inter-provenance also reached extremely significant levels, suggesting that long-term natural selection caused poplars to exhibit high intra-provenance variation in growth and physiological traits, which is similar to that in a related study on cedar [67]. As an important indicator of genetic differentiation among provenances, variance components can be used to further reveal the extent of genetic variation within and between provenances [68,69]. In our study, the percentage of intra-provenance variation for each trait in poplars was higher than 78.36%, and the percentage of inter-provenance variation was less than 21.64%, indicating that the variation for each trait was mainly derived from intra-provenance variation, and inter-provenance variation had less influence on the traits of poplars, which is similar to the results of the previous study conducted by our team [70]. Furthermore, low differentiation in inter-provenances was found in studies of genetic variation in *P. tomentosa* Carrière [71], *P. simonii simonii* [72], and *P. trichocarpa* Torr. & Gray [73], but it differs from the natural *P. euphratica* Oliv. [74]. This may be related to the fact that *P. deltoides* is a heterozygous wind-borne plant, and its provenance sites are mostly distributed in the vast plains and close to the Mississippi, Columbia, and St. Lawrence Rivers. These conditions enhance seed dispersal and facilitate frequent gene exchange among inter-provenances through pollen and seeds, thereby limiting genetic differentiation among inter-provenances to a certain extent [75].

### 3.2. Heritability of Traits in P. deltoides

In general, heritability, as one of the most crucial genetic parameters, reflects the extent of genetic control over plant growth traits. A higher heritability indicates a greater stability of these traits, enhanced parental ability to transmit them, reduced environmental influence, and improved selection effectiveness [76,77]. The degree of genetic control was broadly categorized based on the magnitude of broad-sense heritability: high (>0.80), medium-high (0.60 to 0.79), medium (0.40 to 0.59), and low (<0.40) [55,78]. We found that the heritability of *P. deltoides* provenance traits ranged from 0.36 to 0.77, which is low to medium-high heritability under strong genetic control, which is in line with previous findings [79,80]. *P. deltoides* WUE (0.61 to 0.77) had medium to high heritability among inter-provenances, which was similar to the magnitude of heritability of *P. nigra* L. [81] and *P. trichocarpa* WUE and NUE, suggesting that genetics is the main factor influencing WUE across provenances and genotypes, further supporting the feasibility of early selection for *P. deltoides* provenance and genotypes. However, NUE heritability was much higher in this study than in *P. trichocarpa* [73], either because of differences in plant type or life type [82,83] or because our experimental soil conditions were consistent and, compared to tracking soil δ^15^N in leaves, measuring δ^15^N in leaves is less susceptible to phenotypic plasticity [84]. These results indicate that the materials selected in this study exhibit considerable variation among provenances and genotypes, demonstrating strong heritability and significant potential for genetic improvement and the selection of new cultivars.

### 3.3. Correlation of Traits and Geographic Location in P. deltoides

A correlation analysis responds to the associations that exist between traits and plays an important role in understanding the relationships among different traits [85]. We found that δ^13^C and δ^15^N were significantly and positively correlated with seedling height, and highly significantly and positively correlated with ground diameter and biomass, which indicated that improvements in any of the growth traits would bring positive improvements in WUE and NUE. Notably, higher ground diameter and total biomass resulted in more desirable WUE and NUE genotypes as compared to plant height, which was observed similarly in the study of *P. balsamifera* L. with respect to the covariate trait relationship between growth traits (seedling height) and physiological traits (isotope) [86]. In addition, an extremely significant positive correlation between δ^13^C and δ^15^N was also found in this study, which is consistent with the findings of Chen et al. [87] and Perid et al. [88], which suggests that there is a strong coupling between δ^13^C and δ^15^N driven by water dynamics.

Phenotypic characteristics and physiological traits of plants are often related to the pattern of variations in the geographic latitude and longitude of their growing sites [44,89,90]. In addition, climatic factors can influence C_i_/C_a_ by affecting leaf stomatal conductance (C_i_) and chloroplastic conductance (C_a_), which subsequently drives variability in leaf δ^13^C [91]. Due to high levels of summer light, high temperatures at low latitudes and increased water stress, plants close some stomata to reduce water transpiration losses, which decreases stomatal conductance and intercellular CO_2_ concentration, decreases C_i_/C_a_, and increases δ^13^C [34]. Lower winter temperatures in high-latitude regions decrease leaf sarcolemmal conductance with a decreasing temperature, which increases the resistance to CO_2_ diffusion within the leaf and decreases C_i_/C_a_, leading to an increase in δ^13^C with a decreasing temperature [85,92]. Our findings indicated that the ground diameter, total biomass, and δ^13^C of *P. deltoides* were extremely significantly negatively correlated with latitude, and seedling height, ground diameter, and δ^13^C were extremely significantly positively correlated with longitude. This suggests that their WUE and growth capacity have similar patterns, which may be due to higher WUE and faster growth in low latitude and high longitude provenance areas, and limited energy recharge in high-latitude and low-longitude provenance areas restricts WUE and growth [93]. This result aligns with the climatic characteristics of *P. deltoides* provenance sites. Specifically, the AL provenance site, located at a low latitude, experiences a humid subtropical monsoon climate characterized by abundant rainfall and high levels of summer sunlight. In contrast, the AQ provenance site, situated in a high longitude region, falls within the cool temperate zone and features a humid continental climate with predominantly cloudy and wet weather. In addition, plants are associated with a trade-off between long-term δ^13^C and NUE, with higher δ^13^C coming at the cost of lower NUE [94,95]. However, this trade-off relationship between plant WUE and NUE may be broken under different climatic contexts, showing no significant correlation between the two [96,97]. In the present study, it was similarly found that δ^15^N was not significantly correlated with δ^13^C and latitude and longitude. It is evident that the relationship between WUE and NUE, as well as their respective associations with latitude and longitude, is complex, and influenced by a multitude of factors. Plants may exhibit adaptive changes to adjust water utilization and carbon and nitrogen allocation when faced with environmental stresses. Based on these findings, we conclude that *P. deltoides* from AL and AQ provenances exhibit enhanced growth performance and water acquisition strategies, making them suitable candidates for efficient water utilization.

### 3.4. A Comprehensive Evaluation of P. deltoides

The criteria for selecting suitable materials vary based on different breeding objectives. To achieve the goal of jointly selecting multiple traits, principal component analysis and fuzzy affiliation function methods have been widely employed in the comprehensive evaluation of multiple traits in plants and tree selection studies [98,99]. We utilized a comprehensive index selection focusing on the WUE and NUE of *P. deltoides*, while requiring fast growth and high productivity. The objective was to screen and breed a new generation of *P. deltoides* genotypes suitable for northern China, and to obtain excellent parental materials for Populus hybrid breeding. Based on this method, eight efficient genotypes were obtained in this study, which were from the excellent provenances AQ and AL, among which the two genotypes with the best overall evaluation (178-2-141 and 174-1-2) were from the provenance AQ, and the heritability of each trait from the AQ provenance source was above 0.65. Considering that high heritability does not imply high genetic gain for a specific trait [100]. Genetic gain is a crucial parameter for assessing the effectiveness of breeding, as it reflects the extent to which the breeding population surpasses the existing population, thereby indicating the success of the breeding efforts [101]. Therefore, we evaluated the genetic gain of the selected superior genotypes, and the average genetic gains for seedling height, ground diameter, total biomass, δ^13^C, and δ^15^N were 19.19 cm, 2.01 mm, 9.30 g, 0.458‰, and 1.372‰, respectively. The results align with findings from previous poplar studies [102,103,104], suggesting significant potential for early selection. All superior genotypes exhibited positive genetic gain, which we recommend as promising candidates for subsequent breeding.

## 4. Materials and Methods

### 4.1. Test Materials

**Figure 3:**
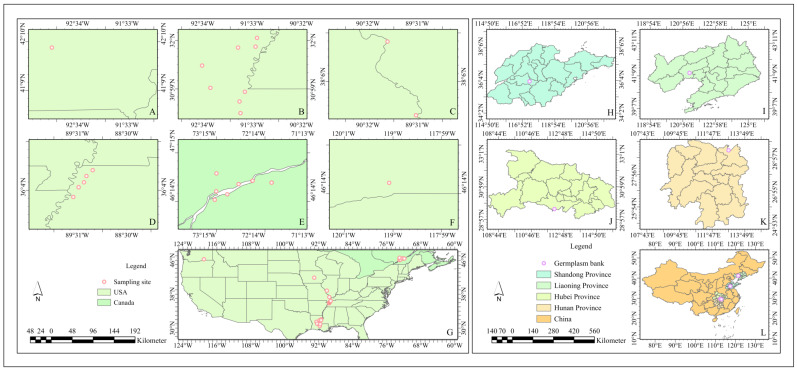
Distribution of provenances and genebanks. The areas marked (**A**–**F**) are the geographical locations of the sampling points of the six provenances of *P. deltoides*: (**A**) Iowa, America (AI); (**B**) Louisiana, America (AL); (**C**) Missouri, America (AM); (**D**) Tennessee, America (AT); (**E**) Quebec, Canada (AQ); and (**F**) Washington, America (AW). The area marked (**G**) is the distribution of the sampling points in the USA and Canada, and the legend, the compass, and the scale are on its left side. The areas marked (**H**–**K**) are the geographical locations of the germplasm resources of *P. deltoides*: (**H**) Ningyang Gaoqiao Forest Farm; (**I**) Daling-river Forest Farm; (**J**) Shishou Poplar Research Institute; (**K**) Junshan Forest Farm. The area marked (**L**) is the distribution of the germplasm resources in China, and the legend, the compass, and the scale are on its left side.

From 2005 to 2009, we collected resources through the phenotypic selection of superior trees within the natural distribution area of *P. deltoides*. Six provenances (Figure 3), 31 families, and 61 clones were finally conserved through selection and seedling multiplication. Between 2008 and 2014, we established genebanks in several climate zones in China, specifically at Junshan Forestry in Yueyang, Hunan; Shishou Poplar Research Institute in Hubei; Gaoqiao Forestry in Ningyang, Tai’an, Shandong; and Dalinghe Forestry in Linghai, Liaoning. The test materials were sourced from these genebanks (Table 8 and Table S1). In April 2016, one-year-old branches were collected, and cuttings were completed in April 2016 in the greenhouse at the Tongzhou Experimental Nursery Base (39°44′01″ N, 116°45′06″ E), under the auspices of the Research Institute of Forestry, Chinese Academy of Forestry. In early August, nine well-grown plants were selected per genotype and subsequently transferred to the field. The field experiment was conducted in a completely randomized block design consisting of three blocks with three replications in each block. The plants received 1200 mL of water every two days and were manually weeded and treated for pests every two weeks.

### 4.2. Trait Measurement

#### 4.2.1. Subsubsection Carbon and Nitrogen Isotope Ratios in Leaves

In September 2016, three replicates of three to five mature functional leaves were collected from each genotype. The leaves were dried at 75 °C and ground, and the ratios of ^13^C to ^12^C and ^15^N to ^14^N in the samples were determined using a DELTA V Advantage isotope ratio mass spectrometer (Thermo Fisher Scientific, Inc., Waltham MA, USA).

#### 4.2.2. Growth Trait

In October 2016, the seedling height, ground diameter, and number of leaves were measured for each genotype. Subsequently, all leaves, roots, and stems were harvested and weighed after being dried at 75 °C until a constant mass was achieved for leaf, stem, and root biomass.

### 4.3. Data Processing

A nested ANOVA, Duncan’s multiple comparisons, correlation analysis, and principal component analysis were performed using SPSS 21.0; a mixed-effects modeling analysis was performed using the R package Asreml 4.0 [105]; and plotting was performed using Origin 2021. The experimental data were recorded using the Excel software application, and the parameters were calculated as follows:

Coefficient of variation formula:

(1) $$\mathrm{CV}=\sigma /\bar{x}\times 100\%,$$

where CV is the coefficient of variation of traits, σ is the standard deviation, and $\bar{x}$ is the mean value.

Total biomass formula:

TB = LN × LB + SB + RB,(2)

where TB is the total biomass, LN is the amount of leave, LB is the single leaf biomass, SB is the stem biomass, and RB is the root biomass.

Carbon/nitrogen isotope abundance formula:

δ^13^C/δ^15^N = (R_Sample_ − RPDB)/RPDB × 1000‰, (3)

where R_Sample_ represents the samples’ ^13^C/^12^C or ^15^N/^14^N ratios, and RPDB is the ^13^C/^12^C, ^15^N/^14^N of the international standard substance PDB (Pee Dee Belemnite); the analytical accuracy of these ratios was ±0.20‰ [34].

Percentage of variation formula:

V_t/s_ = σ^2^_t/s_/(σ^2^_t/s_ + σ^2^_s_), V_s_ = σ^2^_s_/(σ^2^_t/s_ + σ^2^_s_), (4)

where V_t/s_ and V_s_ are the percentages of trait variation in the inter- and intra-provenance, and σ^2^_t/s_ and σ^2^_s_ are the variance components of the inter- and intra-provenance [106].

The fuzzy mathematical affiliation function was used to calculate the composite score of the principal component results:

*y*(PC_*ij*_) = (PC_*ij*_ − PC_*j*min_)/(PC_*j*max_ − PC_*j*min_), (5)

w_*j*_ = r_*j*_/∑r_*j*_, (6)

P_CE−*i*_ = ∑w_*j*_ × y(PC_*ij*_), (7)

where *y*(PC*_ij_*) represents the fuzzy mathematical affiliation function values for the *j*th principal component of the *i*th genotype, PC*_ij_* is the score of the *j*th principal component of the *i*th genotype, PC*_j_*_min_ and PC*_j_*_max_ are the minimum and maximum values of the score of the *j*th principal component, w*_j_* and r*_j_* are the weight and contribution of the *j*th principal component, and P_CE−*i*_ is the composite score of the *i*th genotype [107].

(8) $$Combined\;trait\;superiority\;genotype:\;P_{CE-i}\;\geq \;\frac{\sum_{1}^{256}P_{CE-i}}{256}+2\sigma_{1},$$

where P_CE−*i*_ is the composite score of the *i*th genotype, and σ_1_ is the corresponding standard deviation.

Mixed-effects model for extracting provenance variance components:

y_i_ = μ + S_i_ + R + e_i_, (9)

where y_i_ is the observation of the ith provenance, μ is the mean of all observations, S_i_ is the effect of the provenance, R is the effect of repetition, and e_i_ is the random error. In the model, R indicates fixed effects, and S_i_ indicates random effects.

The heritability of a trait formula:

h^2^ = V_Si_/[V_Si_ + V_ei_], (10)

where h^2^ is the heritability of a trait, V_Si_ is the variance component of the provenance, and V_ei_ is the variance component of the random error [68].

The genetic gain formula:

ΔG = (A_s_−A_p_) × h_i_^2^, (11)

where ΔG is the trait’s genetic gain, A_s_ and A_p_ are the mean values of traits in the selected genotypes and the total population of the experiment, and h_i_^2^ is the heritability of the selected provenance [105].

## 5. Conclusions

*P. deltoides* received its name based on its origin in the Americas. Since its introduction to China, it has been recognized as a widely cultivated species in artificial forests, including timber forests and shelterbelts. However, as its cultivation expands to northern regions—primarily arid and semi-arid areas such as Shandong, Hebei, and Inner Mongolia—its ability to withstand drought and barren conditions gradually diminishes. This decline is evidenced by a significant reduction in survival rates, yield rates, and growth rates. Given the practical challenges and emerging issues, an early screening strategy for provenances and genotypes with optimal resource utilization of *P. deltoides* is particularly important.

Our study revealed significant inter-origin and inter-genotype differences in the growth, water use efficiency, and nutrient use efficiency characteristics of *P. deltoides* germplasm resources, highlighting a high degree of genetic diversity and strong environmental adaptation. Notably, trait variation within provenances emerged as the primary driver of variation, while the low differentiation among provenances suggests that future breeding efforts should prioritize the identification and selection of superior genotypes within these provenances.

The heritability of various traits in *P. deltoides* from the six provenances is generally at a medium to high level, particularly for water and nutrient use efficiency, which are significantly influenced by genetic factors. This suggests a promising potential for cultivating new varieties through genetic improvement. Additionally, the significant positive correlations among the traits of interest indicate that future breeding practices or genetic enhancement efforts could benefit from optimizing single traits, as this may synergistically improve other important traits. Therefore, while focusing on the enhancement of a specific trait in *P. deltoides*, it is essential to consider the comprehensive improvement of other target traits.

Another key result of this study is the successful identification of eight outstanding *P. deltoides* genotypes that demonstrate significant positive genetic gains in growth as well as in the utilization of water and nutrients. Among these, three genotypes originate from Quebec, Canada: 178-2-141, 174-1-2, and 178-2-106. The remaining five genotypes are from Louisiana, USA: LA05-N15, LA05-N25, LA05-N27, LA09-N23, and LA01-N3. These exceptional genotypes represent valuable germplasm resources for the expansion and consolidation of poplar plantations in arid and semi-arid regions globally, including China and their respective provenance areas. Furthermore, they serve as breeding parents for the development of superior new poplar germplasm, thereby establishing a robust material foundation and theoretical support for the ongoing enhancement of poplar genetic improvement.

## Figures and Tables

**Table 1:** Multiple comparisons of growth traits. The data in brackets are the coefficient of variation values. Different lowercase letters indicate significant differences at the 0.05 level. Presented here are Iowa America (AI), Louisiana America (AL), Missouri America (AM), Tennessee America (AT), Quebec Canada (AQ), and Washington America (AW).

Trait	AW	AT	AQ	AM	AL	AI	Mean
Height (cm)	77.67 a (21.27%)	61.56 d (29.91%)	62.49 cd (22.7%)	70.67 b (17.23%)	70.16 b (25.1%)	66.17 c (18.66%)	68.12 (22.48%)
Ground Diameter (mm)	7.38 bc (9.54%)	7.64 b (14.1%)	7.02 d (16.98%)	7.54 b (12.74%)	8.20 a (17.89%)	7.12 cd (10.19%)	7.48 (13.57%)
Total Biomass (g)	20.12 bc (26.49%)	20.67 bc (31.92%)	19.18 c (30.62%)	21.53 b (23.63%)	23.28 a (31.1%)	19.10 c (18.91%)	20.65 (27.11%)

**Table 2:** Analysis of variance of growth traits. ** represents extremely significant correlation (*p* less than 0.01).

Trait	Quadratic Sum		Mean Square		*F*		Percentage of Variation (%)	
	Intra-Provenances	Inter-Provenances	Intra-Provenances	Inter-Provenances	Intra-Provenances	Inter-Provenances	Intra-Provenances	Inter-Provenances
Height (cm)	196,889.5	14,969.44	772.122	2993.89	6.500 **	25.222 **	90.53	9.47
Ground Diameter (mm)	1142.81	136.64	4.48	27.33	6.29 **	38.34 **	83.48	16.52
Total Biomass (g)	29,099.52	1597.38	114.12	319.48	5.28 **	14.79 **	93.75	6.25

**Table 3:** WUE and NUE of *P. deltoides*. Different lowercase letters indicate significant differences at the 0.05 level.

Trait	AW	AT	AQ	AM	AL	AI	Mean
δ^13^C (‰)	−30.277 b	−29.937 a	−30.514 c	−30.555 c	−29.894 a	−29.804 a	−30.164
	(1.84%)	(2.48%)	(2.35%)	(1.90%)	(2.26%)	(1.81%)	(2.11%)
δ^15^N (‰)	−0.798	−0.916	−0.967	−0.814	−0.834	−0.656	−0.831
	(97.90%)	(88.36%)	(114.89%)	(85.54%)	(91.49%)	(91.24%)	(94.90%)

**Table 4:** Analysis of variance of WUE and NUE. ** represents extremely significant correlation (*p* less than 0.01).

Trait	Quadratic Sum		Mean Square		*F*		Percentage of Variation (%)	
	Intra- Provenances	Inter- Provenances	Intra- Provenances	Inter- Provenances	Intra- Provenances	Inter- Provenances	Intra- Provenances	Inter- Provenances
δ^13^C	428.19	69.29	1.68	13.86	9.85 **	81.31 **	78.36	21.64
δ^15^N	1055.17	6.27	4.14	1.25	5.51 **	1.67	99.49	0.51
Mean							88.93	11.08

**Table 5:** Heritability of traits.

Heritability (h^2^)	Height	Ground Diameter	Total Biomass	WUE	NUE
AM	0.36	0.49	0.48	0.62	0.60
AI	0.71	0.60	0.55	0.73	0.42
AW	0.61	0.47	0.42	0.73	0.39
AQ	0.69	0.67	0.66	0.77	0.71
AL	0.51	0.58	0.48	0.69	0.66
AT	0.68	0.51	0.50	0.61	0.32

**Table 6:** Principal component analysis of traits. Presented here are principal component 1 (PC_1_), principal component 2 (PC_2_), carbon isotope composition (δ^13^C), and nitrogen isotope composition (δ^15^N).

Trait	Total Biomass	Ground Diameter	Height	δ^15^N	δ^13^C	Eigenvalue	Contribution Rate/%	Cumulative Contribution Rate/%
PC_1_	0.94	0.90	0.88	0.37	0.40	2.76	55.10	55.10
PC_2_	−0.15	−0.19	−0.27	0.73	0.70	1.14	22.91	78.01
Comprehensive evaluation model: P_CE−*i*_ = 0.707 × y(PC_*i*1_) + 0.293 × y(PC_*i*2_)								

**Table 7:** Comprehensive evaluation of superior genotypes. Presented here are Louisiana America (AL) and Quebec Canada (AQ).

Genotype Number	Population	Comprehensive Score	Ranking	Genetic Gain (ΔG)				
				Height (cm)	Ground Diameter (mm)	Total Biomass (g)	WUE (‰)	NUE (‰)
178-2-141	AQ	0.81	1	22.17	3.85	16.40	0.221	1.265
174-1-2	AQ	0.80	2	23.09	1.41	8.86	0.721	3.145
LA05-N15	AL	0.77	3	23.53	1.82	7.49	0.852	1.104
178-2-106	AQ	0.76	4	27.46	2.80	15.78	0.008	1.167
LA05-N25	AL	0.71	5	22.34	1.48	7.96	0.532	0.807
LA05-N27	AL	0.70	6	9.93	1.44	4.89	0.774	1.374
LA09-N23	AL	0.67	7	11.46	1.60	6.08	0.110	1.499
LA01-N3	AL	0.65	8	13.50	1.66	6.94	0.447	0.615
Mean				19.19	2.01	9.30	0.458	1.372

**Table 8:** Provenances information of *P. deltoides*.

Provenance	Longitude (W)	Latitude (N)	Type of Climate	Genotype Number
Iowa, America (AI)	93°05′60″	41°52′48″	Temperate continental climate	19
Louisiana, America (AL)	91°52′48″	31°18′36″	Subtropical humid climate	37
Missouri, America (AM)	89°50′24″	38°03′36″	Subtropical humid climate	11
Tennessee, America (AT)	89°24′00″	36°09′36″	Subtropical humid climate	51
Quebec, Canada (AQ)	72°29′24″	46°20′24″	Temperate continental climate	124
Washington, America (AW)	119°04′48″	46°13′12″	Temperate continental climate	14
			Total	256

## Data Availability

The data underlying this article are available in the article and in its Supplementary Materials.

## Supplementary Materials

The following supporting information can be downloaded at: https://www.mdpi.com/article/10.3390/plants13162228/s1, Table S1: Information of provenances and genotypes of *P. deltoides*; Table S2: Clonal character ranking of *P. deltoides*.
