# Supplementary material for: Genetic Evaluation of Water Use Efficiency and Nutrient Use Efficiency in Populus deltoides Bartr. ex Marsh. Seedlings in China
Source: Plants (Basel). 2024 Aug 11;13(16):2228. doi: 10.3390/plants13162228 (PMC11359723; doi:10.3390/plants13162228)
Supplement: Supplementary file 1 [file plants-13-02228-s001.zip › plants-3111329-supplementary.pdf]

Supplementary Materials:

Table S1. Information of provenances and genotypes of *P. deltoides*.

Number	Provenance	Genotype number	Number	Provenance	Genotype number	Number	Provenance	Genotype number	Number	Provenance	Genotype number
1	AM	484	65	AQ	174-3-4	129	AQ	178-2-195	193	AL	LA07-N35
2	AM	489	66	AQ	174-3-5	130	AQ	178-2-198	194	AL	LA07-N45
3	AM	491	67	AQ	174-3-6	131	AQ	179-1	195	AL	LA07-N55
4	AM	494	68	AQ	174-3-7	132	AQ	179-2	196	AL	LA07-N6
5	AM	495	69	AQ	174-3-8	133	AQ	179-3	197	AL	LA08-N1
6	AM	499	70	AQ	174-3-10	134	AQ	179-4	198	AL	LA08-N21
7	AM	500	71	AQ	174-3-11	135	AQ	179-6	199	AL	LA08-N3
8	AM	501	72	AQ	174-3-12	136	AQ	179-7	200	AL	LA08-N4
9	AM	503	73	AQ	174-3-13	137	AQ	179-9	201	AL	LA08-N52
10	AM	504	74	AQ	174-3-15	138	AQ	179-10	202	AL	LA09-N1
11	AM	508	75	AQ	175-1-1	139	AQ	180-1	203	AL	LA09-N23
12	AI	5006	76	AQ	175-1-2	140	AQ	180-3	204	AL	LA09-N31
13	AI	5007	77	AQ	175-1-3	141	AQ	180-4	205	AL	LA09-N59
14	AI	5008	78	AQ	175-1-4	142	AQ	180-8	206	AT	TN01-27
15	AI	5009	79	AQ	176-3-2	143	AQ	180-9	207	AT	TN01-54
16	AI	5010	80	AQ	176-3-3	144	AQ	180-11	208	AT	TN01-65
17	AI	5012	81	AQ	177-3-8	145	AQ	180-12	209	AT	TN01-71
18	AI	5013	82	AQ	177-3-9	146	AQ	180-16	210	AT	TN01-79
19	AI	5014	83	AQ	178-2-7	147	AQ	180-17	211	AT	TN01-88
20	AI	5015	84	AQ	178-2-8	148	AQ	180-18	212	AT	TN01-92
21	AI	5036	85	AQ	178-2-9	149	AQ	180-25	213	AT	TN01-N43

22	AI	5037	86	AQ	178-2-15	150	AQ	180-28	214	AT	TN01-N50
23	AI	5038	87	AQ	178-2-16	151	AQ	180-30	215	AT	TN01-N58
24	AI	5039	88	AQ	178-2-17	152	AQ	180-31	216	AT	TN02-112
25	AI	5040	89	AQ	178-2-18	153	AQ	180-34	217	AT	TN02-117
26	AI	5041	90	AQ	178-2-21	154	AQ	180-36	218	AT	TN02-16
27	AI	5042	91	AQ	178-2-29	155	AQ	180-38	219	AT	TN02-34
28	AI	5043	92	AQ	178-2-31	156	AQ	180-39	220	AT	TN02-5
29	AI	5044	93	AQ	178-2-35	157	AQ	180-40	221	AT	TN02-63
30	AI	5045	94	AQ	178-2-36	158	AQ	180-41	222	AT	TN02-88
31	AW	5017	95	AQ	178-2-38	159	AQ	180-45	223	AT	TN02-9
32	AW	5018	96	AQ	178-2-39	160	AQ	180-46	224	AT	TN02-N46
33	AW	5019	97	AQ	178-2-40	161	AQ	180-52	225	AT	TN02-N57
34	AW	5020	98	AQ	178-2-43	162	AQ	181-1	226	AT	TN03-N10
35	AW	5021	99	AQ	178-2-56	163	AQ	181-2	227	AT	TN03-N22
36	AW	5022	100	AQ	178-2-57	164	AQ	182-2	228	AT	TN03-N27
37	AW	5023	101	AQ	178-2-58	165	AQ	182-3	229	AT	TN03-N28
38	AW	5025	102	AQ	178-2-59	166	AQ	182-4	230	AT	TN03-N34
39	AW	5027	103	AQ	178-2-69	167	AQ	183-1	231	AT	TN03-N49
40	AW	5030	104	AQ	178-2-82	168	AQ	183-2	232	AT	TN03-N5
41	AW	5031	105	AQ	178-2-88	169	AL	LA01-N2	233	AT	TN03-N50
42	AW	5032	106	AQ	178-2-94	170	AL	LA01-N3	234	AT	TN03-N51
43	AW	5034	107	AQ	178-2-98	171	AL	LA01-N30	235	AT	TN03-N55
44	AW	5035	108	AQ	178-2-106	172	AL	LA03-N1	236	AT	TN03-N59
45	AQ	174-1-2	109	AQ	178-2-110	173	AL	LA03-N3	237	AT	TN03-N7
46	AQ	174-1-3	110	AQ	178-2-114	174	AL	LA04-N19	238	AT	TN04-13
47	AQ	174-1-4	111	AQ	178-2-122	175	AL	LA04-N49	239	AT	TN04-15

48	AQ	174-1-5	112	AQ	178-2-123	176	AL	LA04-N53	240	AT	TN04-22
49	AQ	174-1-6	113	AQ	178-2-124	177	AL	LA04-N56	241	AT	TN04-29
50	AQ	174-1-7	114	AQ	178-2-125	178	AL	LA05-N15	242	AT	TN04-N32
51	AQ	174-1-8	115	AQ	178-2-141	179	AL	LA05-N19	243	AT	TN04-N46
52	AQ	174-1-9	116	AQ	178-2-149	180	AL	LA05-N25	244	AT	TN05-N13
53	AQ	174-1-12	117	AQ	178-2-153	181	AL	LA05-N27	245	AT	TN05-N14
54	AQ	174-1-13	118	AQ	178-2-157	182	AL	LA05-N39	246	AT	TN05-N21
55	AQ	174-1-14	119	AQ	178-2-160	183	AL	LA05-N5	247	AT	TN05-N4
56	AQ	174-1-15	120	AQ	178-2-162	184	AL	LA05-N50	248	AT	TN05-N41
57	AQ	174-1-17	121	AQ	178-2-164	185	AL	LA05-N6	249	AT	TN05-N46
58	AQ	174-2-1	122	AQ	178-2-165	186	AL	LA06-N12	250	AT	TN05-N47
59	AQ	174-2-3	123	AQ	178-2-171	187	AL	LA06-N27	251	AT	TN05-N48
60	AQ	174-2-5	124	AQ	178-2-172	188	AL	LA06-N30	252	AT	TN05-N49
61	AQ	174-2-7	125	AQ	178-2-180	189	AL	LA06-N41	253	AT	TN05-N53
62	AQ	174-3-1	126	AQ	178-2-184	190	AL	LA07-N10	254	AT	TN05-N59
63	AQ	174-3-2	127	AQ	178-2-186	191	AL	LA07-N28	255	AT	TN05-N60
64	AQ	174-3-3	128	AQ	178-2-190	192	AL	LA07-N30	256	AT	TN05-N8

Table S2. Clonal character ranking of *P. deltoides*.

Trait	Genotype number	Mean ± standard deviation	Provenance	Ranking	Trait	Genotype number	Mean ± standard deviation	Provenance	Ranking
Height /cm	LA05-N15	111.00±3.00	AL	1	$\delta^{13}C$/‰	LA08-N52	-28.168±0.29	AL	1
	LA05-N25	108.67±6.11	AL	2		LA08-N21	-28.240±0.51	AL	2
	TN01-92	106.67±10.21	AT	3		TN05-N4	-28.296±0.74	AT	3
	178-2-106	104.67±15.57	AQ	4		TN02-N46	-28.314±0.44	AT	4
	174-1-2	98.33±1.53	AQ	5		179-2	-28.794±0.49	AQ	5
	5023	98.00±3.61	AW	6		178-2-88	-28.804±0.09	AQ	6
	TN02-112	97.33±21.22	AT	7		TN01-N58	-28.831±0.32	AT	7
	178-2-141	97.00±32.42	AQ	8		174-3-5	-28.832±0.03	AQ	8
	LA06-N12	94.67±16.20	AL	9		5036	-28.856±0.82	AI	9
	LA08-N1	94.33±25.50	AL	10		TN02-N57	-28.934±0.99	AT	10
Ground Diameter /mm	178-2-141	13.11±2.20	AQ	1	$\delta^{15}N$/‰	491	4.200±1.36	AM	1
	178-2-106	11.54±0.86	AQ	2		179-6	3.596±1.81	AQ	2
	174-1-6	10.84±0.67	AQ	3		174-1-2	3.531±0.46	AQ	3
	LA05-N15	10.50±1.03	AL	4		178-2-184	1.659±0.09	AQ	4
	LA01-N3	10.22±1.84	AL	5		178-2-58	1.624±3.59	AQ	5
	LA06-N12	10.18±1.03	AL	6		LA09-N23	1.373±0.16	AL	6
	LA07-N6	10.13±1.83	AL	7		5044	1.314±0.44	AI	7
	LA09-N23	10.12±0.76	AL	8		LA05-N27	1.184±1.26	AL	8
	LA05-N25	9.91±1.62	AL	9		175-1-2	0.990±1.38	AQ	9
	LA05-N27	9.85±1.64	AL	10		TN02-5	0.982±0.44	AT	10
Total Biomass /g	178-2-141	45.06±15.71	AQ	1	Total Biomass /g	TN02-112	34.53±8.81	AT	7
	178-2-106	44.12±6.02	AQ	2		174-1-2	33.64±5.14	AQ	8

LA05-N25	36.80±10.91	AL	3	TN05-N8	33.44±2.20	AT	9
LA05-N15	35.82±1.37	AL	4	TN05-N14	33.38±4.80	AT	10
LA07-N6	35.81±9.63	AL	5	LA08-N1	33.06±11.27	AL	11
LA01-N3	34.67±13.68	AL	6	LA09-N23	32.89±7.74	AL	12

[1] The underline indicates that the genotype has two or more excellent single traits.
